# Argon Microwave Plasma Processed Electrodeposited FeCo Catalysts on Ti Paper as an Anode Porous Transport Layer (PTL) for Cathode-Dry Anion Exchange Membrane Water Electrolysis (AEMWE)

**DOI:** 10.1021/acsomega.5c09854

**Published:** 2026-03-11

**Authors:** Hsing-Chen Wu, Shuo-En Yu, I-Chun Cheng, Jian-Zhang Chen

**Affiliations:** † Institute of Applied Mechanics, 33561 National Taiwan University, Taipei City 106319, Taiwan; ‡ Graduate School of Advanced Technology, National Taiwan University, Taipei City 106319, Taiwan; § Graduate Institute of Photonics and Optoelectronics and Department of Electrical Engineering, National Taiwan University, Taipei City 106319, Taiwan; ∥ Advanced Research Center for Green Materials Science and Technology, National Taiwan University, Taipei City 106319, Taiwan; ⊥ Research Center for Applied Sciences, Academia Sinica, Taipei City 115201, Taiwan

## Abstract

ED-FeCo/TP is synthesized by the electrochemical deposition of FeCo on titanium fiber paper (TP) as the catalyst for the oxygen evolution reaction (OER) in cathode-dry operation anion exchange membrane water electrolysis (AEMWE). With 15 s Ar microwave plasma (ArMP) treatment, the catalytic performance is enhanced. The ED-FeCo/TP treated with ArMP (ED-FeCo/TP-ArMP15) exhibits an overpotential of 459 mV@100 mA/cm^2^ and an improved Tafel slope of 211.1 mV/dec, a higher double-layer capacitance (2.80 mF/cm^2^), a lower charge transfer resistance (1.10 Ω), and enhanced electrochemical activity. ArMP treatment provides a reduction effect, leading to the formation of abundant metallic Fe^0^ and Co^0^ in ED-FeCo/TP-ArMP15. The cathode-dry AEMWE system with ArMP treatment (Ru/CP(−)//ED-FeCo/TP-ArMP15­(+)) exhibits better performance at higher current densities than the system without ArMP treatment (Ru/CP(−)//ED-FeCo/TP­(+)). When applied as the anode in a cathode-dry AEMWE system operated at 70 °C, the cell voltage at a current density of 500 mA/cm^2^ decreases from 1.88 V for ED-FeCo/TP to 1.79 V for ED-FeCo/TP-ArMP15. These results indicate that ArMP treatment effectively enhances the OER efficiency.

## Graphical abstract

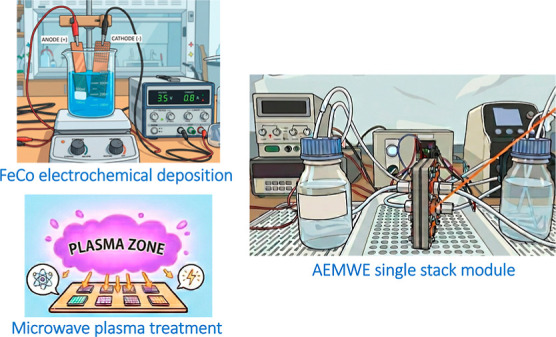

## 1 Introduction

With the increasing global demand for energy, the search for clean and sustainable alternatives has become one of the most important research directions.^1^ Among various renewable options, hydrogen energy is considered a highly promising technology because hydrogen can serve as a clean energy carrier that is both storable and transportable.^2–5^ In the development of hydrogen energy, hydrogen production technologies play a key role.

Among the various hydrogen production methods, water electrolysis is recognized as one of the most efficient and sustainable routes. Through simple system configurations, hydrogen can be produced via the hydrogen evolution reaction (HER) and the oxygen evolution reaction (OER).^6–8^ When powered by renewable energy sources, this process can generate hydrogen with low carbon emissions, commonly referred to as green hydrogen.^2,7^

Electrochemical water electrolysis technologies include alkaline water electrolysis (AWE), proton exchange membrane water electrolysis (PEMWE), and anion exchange membrane water electrolysis (AEMWE). AEMWE integrates the benefits of both AWE and PEMWE, operating in a relatively noncorrosive environment, adopting nonprecious metal catalysts and producing high-purity hydrogen.^3,6,7,9–12^ However, many challenges still need to be overcome in the development of AEMWE. In water electrolysis systems, the OER is considered the important step because it involves a four-electron transfer process, which is relatively sluggish and limits the overall hydrogen production efficiency.^10,13,14^ Therefore, the development of efficient OER electrocatalysts and porous transport layers (PTLs) for the anode is crucial.^13,15–18^ Currently, noble-metal-based catalysts such as IrO_2_ and RuO_2_ exhibit excellent catalytic performance, but their high cost severely restricts large-scale application in water electrolysis, posing a bottleneck for system development.^13,19^ In contrast, nonprecious transition metals such as Ni, Fe, and Co have attracted significant attention due to their favorable electronic structures, good OER activity, and earth abundance, offering a cost advantage.^20–26^ Among them, FeCo-based materials have demonstrated remarkable OER performance. The synergistic interaction between Fe and Co can effectively tune the electronic structure and adsorption energy of the catalyst, facilitating the formation of highly active phases with superior OER catalytic performance.^22,27^ Therefore, FeCo-based materials are considered highly promising candidates for developing efficient, low-cost OER catalysts. On the other hand, in PTL development, Ti has shown advantages as a substrate material owing to its excellent mechanical strength, thermal conductivity, and corrosion resistance, particularly under the harsh electrochemical environments encountered in water electrolysis systems.^28^

Recently, plasma surface and interface modification technologies have been reported to offer rapid, cost-effective, and versatile strategies for improving the electrochemical performance of electrocatalysts and energy storage materials.^29–34^ Plasma, consisting of ionized gas molecules, contains a variety of energetic species such as free radicals, electrons, and ions.^20,35^ These highly reactive species can modify material surfaces by altering surface functional groups, wettability, bonding types, and defects, thereby enabling controllable and targeted material design.^30,31,33,35,36^ Among various plasma systems, microwave plasma (MP) is particularly advantageous because microwave energy can be directly coupled to the gas phase, generating high-intensity plasma with a high dissociation rate.^17,37,38^ This makes it especially suitable for rapid surface modification and MP has also been widely applied in semiconductor and chemical engineering industries.^39,40^

This study focuses on AEMWE and adopts the cathode-dry operation mode, in which water is transported from the anode to the cathode via the AEM. Some studies have shown that, compared to the dual-feed mode, cathode-dry operation improves the purity of the generated hydrogen.^41–45^ Previously we have demonstrated improved performance of oxygen evolution reaction for AEMWE by using atmospheric pressure plasma jet processed NiMoO_4_, NiFe, and NiCo metal–organic framework electrocatalysts.^20,21,30^ Furthermore, MP is capable of oxidizing and reducing of NiCo metal–organic framework materials, depending on the types of plasma gases.^35^ Recently, we also have shown that MP can be used for enhancing the electrocatalytic performance of self-catalytic stainless steel fiber paper porous transport layer for duel-wet and cathode-dry AEMWE.^17^ This study investigates the use of Ti fiber paper (TP) as a substrate material for the PTL in the anode of AEMWE. FeCo materials are grown on TP via electrochemical deposition, following which surface treatment is performed using MP. The experimental data indicate that FeCo electrocatalysts show improved performance with 15 s ArMP processing.

## 2 Experimental Section

### 2.1 Pretreatment of TP

During the pretreatment process, a 6 cm × 5 cm TP substrate was cleaned using ultrasonication to remove surface impurities and improve hydrophilicity. The substrate was sequentially cleaned with a 3 M solution of hydrochloric acid, ethanol and deionized water for 15 min each.

### 2.2 Preparation of FeCo/TP

After pretreatment, TP underwent electrochemical deposition of Fe- and Co-based materials. The ED solution was prepared as follows: 1.4 g of ferric nitrate nonahydrate (Fe­(NO_3_)_3_·9H_2_O) and 1 g of cobalt nitrate hexahydrate (Co­(NO_3_)_2_·6H_2_O) were added to 150 mL of ethanol and continuously stirred until the solution was clear.

As shown in Figure 1, a 6 cm × 5 cm TP substrate was immersed in the prepared plating solution, with a submerged area of 5 cm × 5 cm, and the unimmersed portion was connected to the negative terminal of the electrode. Simultaneously, a 6 cm × 5 cm carbon paper (CP, CeTech.) was employed as the inert electrode; it was also immersed in the solution with an active area of 5 cm × 5 cm, and connected to the positive terminal of the electrode. The deposition process was carried out under a constant current of 0.2 A for a duration of 20 min. Upon the completion of electroplating, the FeCo-coated TP substrate was removed, air-dried at ambient temperature for 1 h, and further dried in an oven at 60 °C for an additional 1 h. This procedure resulted in the formation of the ED-FeCo/TP sample. The sample treated with ArMP for 15 s was named ED-FeCo/TP-ArMP15.

**Figure 1:**
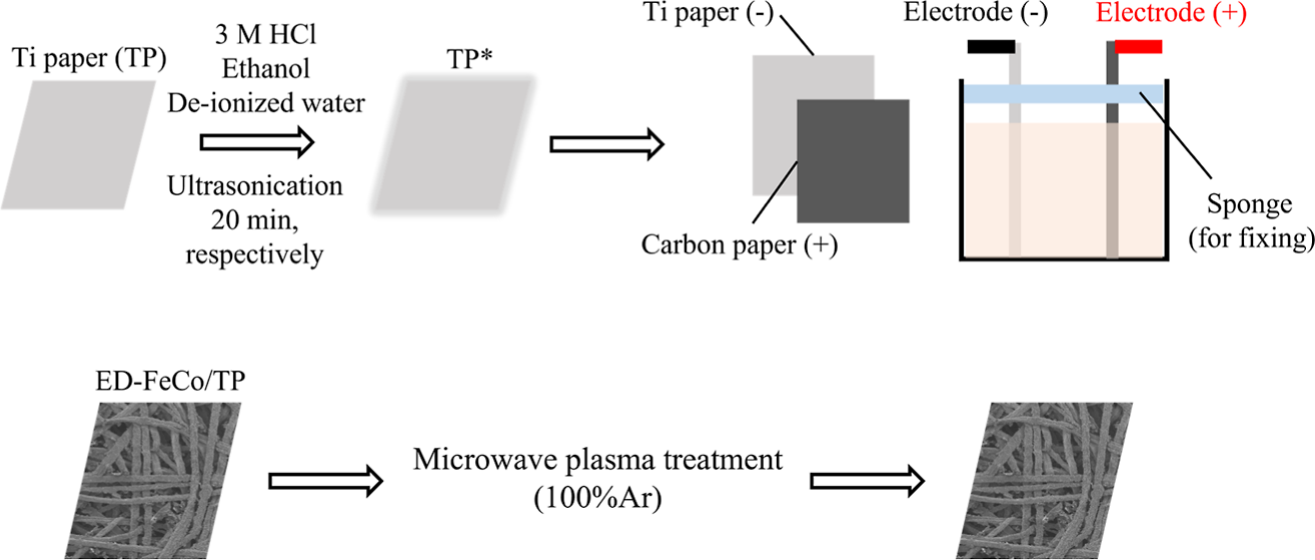
Schematic illustration of synthesis process of ED-FeCo/TP and ED-FeCo/TP-ArMP15.

### 2.3 MP Treatment

Figure 2 presents a schematic representation of the MP system. The experimental setup includes a switch-mode power supply (SM745, Richardson Electronics, Ltd.) linked to an MH2.0W-S microwave head capable of delivering adjustable microwave power with a maximum output of 2.0 kW at a frequency of 2.45 GHz to achieve precise energy regulation. During the processing, Ar gas (20 sccm) was introduced through the gas inlet to generate plasma, with the plasma power set at 280 W and a fixed treatment duration of 15 s. In this study, the samples underwent ArMP treatment, with all treatment times fixed at 15 s. To investigate the impact of various treatments, the samples were classified into three distinct groups for comparative analysis: untreated TP, electroplated ED-FeCo/TP, and MP-treated ED-FeCo/TP-ArMP15.

**Figure 2:**
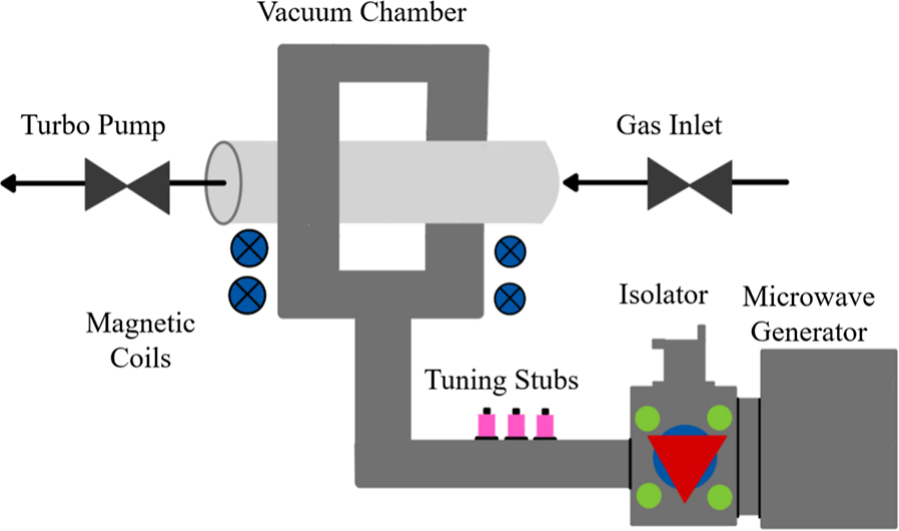
Schematic diagram of MP system.

### 2.4 Material Characterization

X-ray diffraction (XRD) analysis was conducted using a Bruker D2 PHASER XE-T XRD system equipped with Cu Kα radiation to examine the crystal structure. The water contact angle was measured using a goniometer (Sindatek, model 100SB). The morphology and elemental composition of the material were analyzed using scanning electron microscopy (SEM, JEOL 6500F) combined with energy-dispersive X-ray spectroscopy (EDS, Oxford Instruments, AZtecOne, UK). X-ray photoelectron spectroscopy (XPS, Sigm a Probe, Thermo VG Scientific, Waltham, MA, USA) was used to determine the quantitative chemical composition and bonding states of the material.

### 2.5 Synthesis of Ru/CP Electrocatalyst

AEMWE was employed as the testing platform to evaluate the performance of ED-FeCo/TP and ED-FeCo/TP-ArMP15 as anode electrodes, with Ru/CP serving as the cathode electrode in both cases. Ru/CP was synthesized via a solvothermal method. Specifically, 0.468 g of RuCl_3_ was solubilized in a mixed solution of 20 mL ethylene glycol and 20 mL ethanol to prepare the precursor solution. A carbon paper substrate (2.3 cm × 2.3 cm) was placed in a stainless steel autoclave with a polytetrafluoroethylene (PTFE) lining and exposed to a temperature of 160 °C in an oven for 16 h. After cooling to ambient temperature, the sample was ultrasonically cleaned with deionized water and subsequently dried at 60 °C to yield the Ru/CP catalyst.

### 2.6 Electrochemical Measurements

The electrochemical performance was thoroughly investigated using an Autolab electrochemical workstation (PGSTAT204, Metrohm, Utrecht, The Netherlands) to ensure a systematic analysis. All measurements were conducted in a 1 M KOH electrolyte with a three-electrode configuration, in which a platinum electrode functioned as the counter electrode (CE), an Ag/AgCl electrode was used as the reference electrode (RE), and ED-FeCo/TP and ED-FeCo/TP-ArMP15 served as the working electrodes (WE). In this study, the potential is referenced to the reversible hydrogen electrode (RHE) (eq 1)^17,20,46^

1 $$E_{RHE}=E_{Ag/AgCl}+0.059\times pH+0.197$$

The OER activity was evaluated by recording polarization curves via linear sweep voltammetry (LSV) at a scan rate of 5 mV/s, and the Tafel slope values were determined. The following equation calculates the overpotential (η) for the OER (eq 2)^46^

2 $$\eta =E_{RHE}-1.23\;V$$

To eliminate the resistance arising from the electrolyte and the three-electrode configuration setup, IR compensation was applied to correct the LSV polarization curves. The compensation was performed according to the following equation^47,48^

3 $$E_{corrected}=E_{measured}-0.9\cdot I\cdot R_{solution}$$

where (*E*_measured_) is the measured potential. After conversion, the potential corrected with 90% IR compensation (*E*_corrected_) was obtained by subtracting the product of the measured current (*I*) and the solution resistance (*R*_solution_) from the measured potential.

Cyclic voltammetry (CV) was performed within the non-Faradaic region at scan rates of 20–300 mV/s. The electrochemical double-layer capacitance (2*C*_dl_) was calculated from the CV curves. Furthermore, electrochemical impedance spectroscopy (EIS) was performed at a 400 mV overpotential (with respect to RHE) across a frequency range of 100 kHz to 0.1 Hz.

### 2.7 AEMWE Test

To evaluate the practical application of the electrocatalyst in an AEMWE, a series of performance tests and stability analyses were conducted. Figure 3 shows schematic representation of the water electrolysis system. The electrolyzer uses a commercial module from Dioxide Materials Inc., which consists of a bipolar plate, a PTFE gasket, and a heating plate integrated with a catalyst-coated PTL. The catalyst-coated PTLs were configured with ED-FeCo/TP and MP-treated ED-FeCo/TP-ArMP15 as the anode materials for comparison, in order to investigate the effect of MP treatment on the electrocatalyst properties. The cathode used Ru/CP, with the anode and cathode separated by an AEM (Fumasep FAA-3-50, Fuel Cell Store).

**Figure 3:**
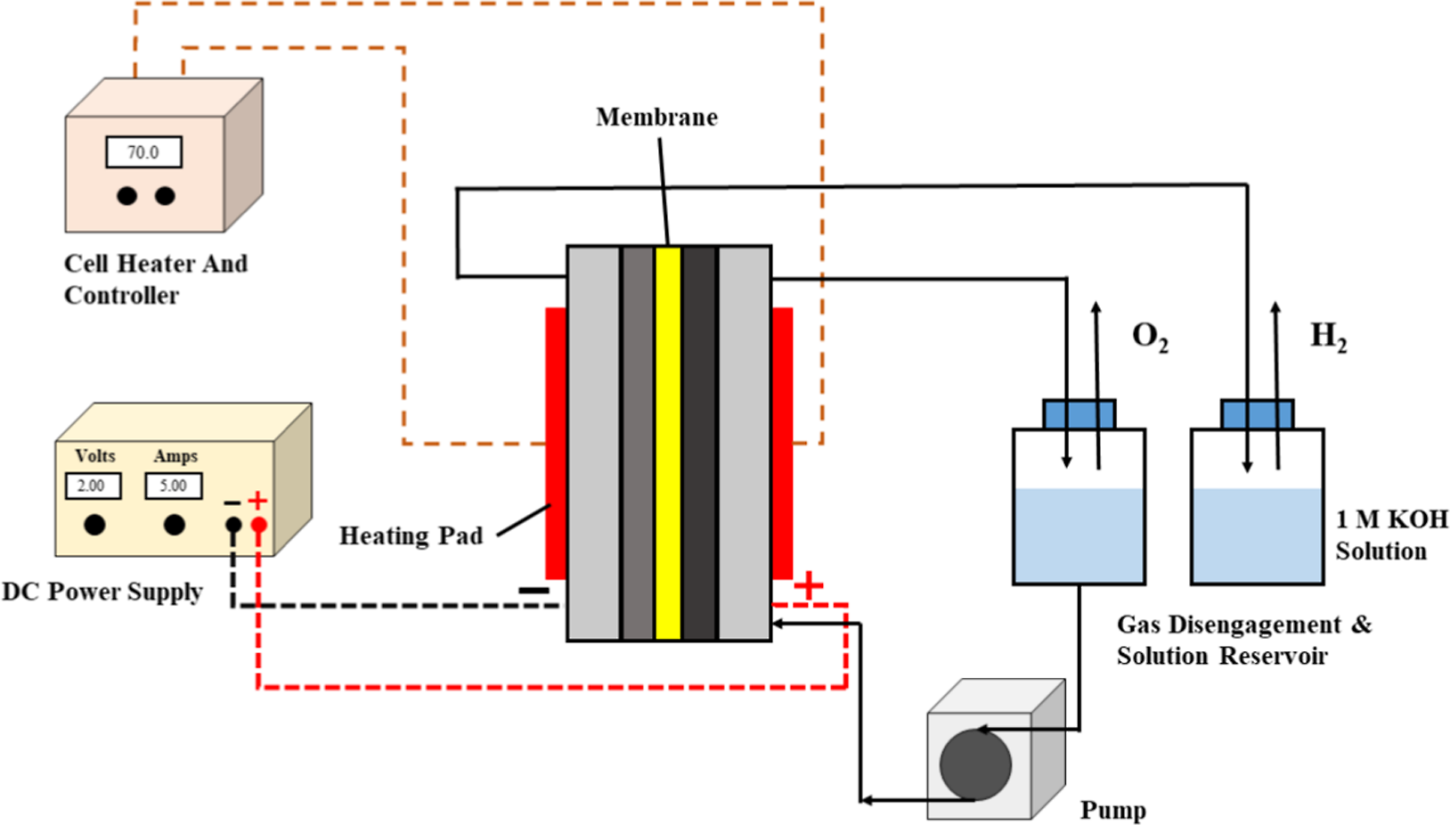
Schematic diagram of AEMWE system.

The performance was tested at 25 °C, 50 °C, and 70 °C. Temperature control was achieved through heating pads on both sides, and precise regulation was provided by a PID controller (DTA4848 V1, Delta Electronics, Taipei, Taiwan) in conjunction with a K-type thermocouple for accurate measurement and adjustment for temperature. The performance under different temperature conditions was evaluated by calculating the energy efficiency and specific energy consumption, to assess the impact of MP treatment on the AEMWE performance.

## 3 Results and Discussion

### 3.1 Water Contact Angle

The surface hydrophilicity of materials plays a crucial role in determining the performance of both electrocatalysts and PTL. Hydrophilic materials facilitate the detachment of gas bubbles generated during electrolysis reactions, allowing for better contact between the electrode and the electrolyte and resulting in lower interfacial resistance.^49,50^ Therefore, good hydrophilicity has a positive effect on electrocatalytic reactions. Figure 4a–c illustrate the water contact angles of TP, ED-FeCo/TP, and ED-FeCo/TP-ArMP15, respectively. As shown in Figure 4a, the untreated TP exhibits a water contact angle of approximately 104.75°, indicating its hydrophobic nature. After electrochemical deposition of FeCo, the resulting ED-FeCo/TP sample (Figure 4b) demonstrates a reduced water contact angle of approximately 81.98°, maintaining a hydrophobic surface. In contrast, the ArMP-treated ED-FeCo/TP-ArMP15 sample (Figure 4c) exhibited a dramatic change in hydrophilicity, where the water droplet is fully absorbed within 0.1 s. These results demonstrate that MP treatment significantly enhances the hydrophilicity of the catalyst surface. The change in hydrophilicity may originate from the interaction between the reactive species and energetic particle bombardment within the plasma, which alters the surface energy of the material.^51–53^ In addition, energetic ions and radicals introduced by the plasma can generate hydrophilic functional groups on the surface, thereby enhancing the hydrophilic nature of the material.^51–54^

**Figure 4:**
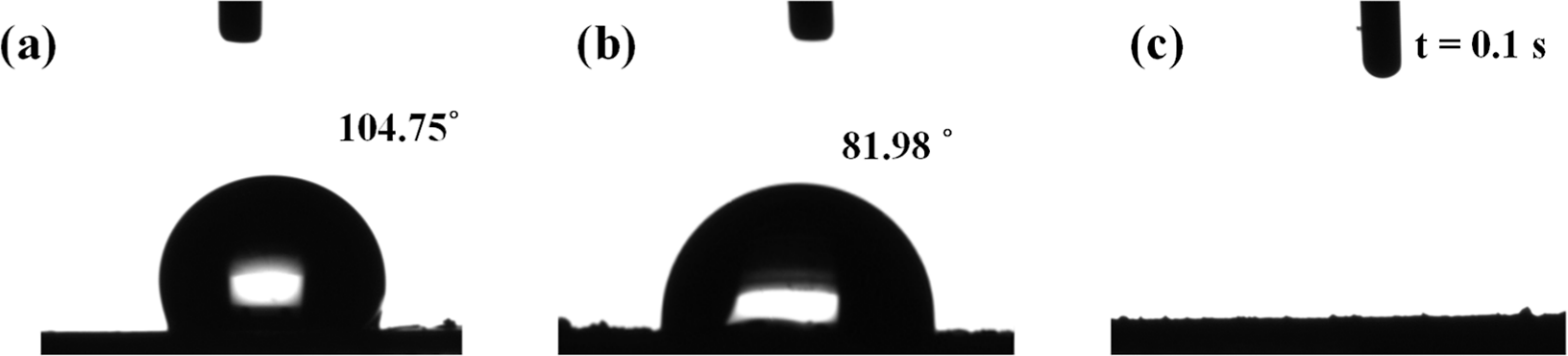
Water contact angle measurement results of (a) TP, (b) ED-FeCo/TP and (c) ED-FeCo/TP-ArMP15.

### 3.2 SEM

Figure 5 shows SEM images of TP, ED-FeCo/TP, and ED-FeCo/TP-ArMP15, illustrating the surface morphologies of the materials and the influence of plasma treatment. Figure 5a shows the magnified SEM image of TP, revealing a relatively smooth surface morphology. In contrast, Figure 5b illustrates the surface structure of ED-FeCo/TP, prepared via the electrochemical deposition of FeCo, which displays aggregated granular features corresponding to the deposited FeCo materials.

**Figure 5:**
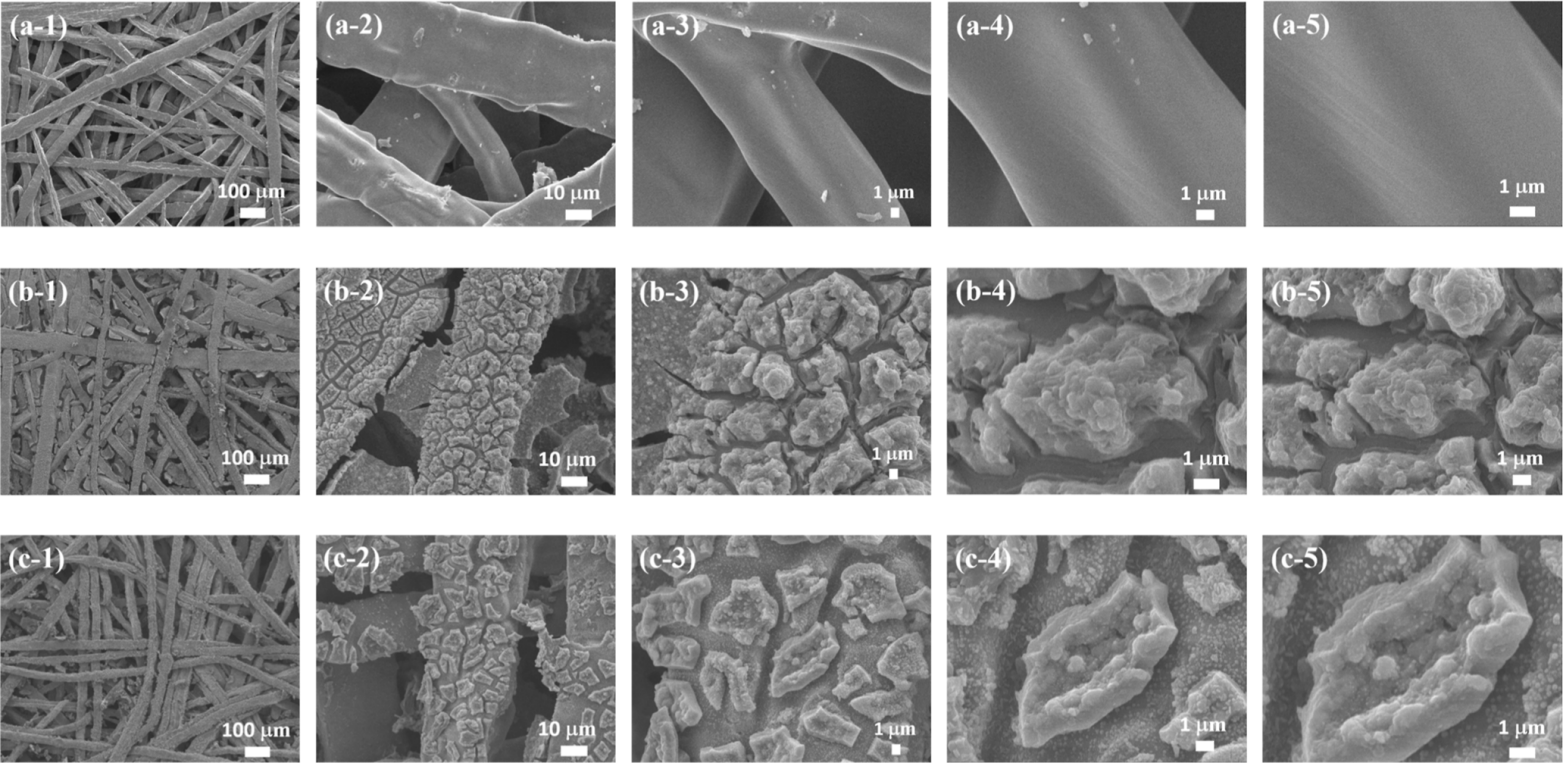
SEM micrographs of TP at magnifications of (a-1) 100×, (a-2) 1000×, (a-3) 3000×, (a-4) 7000×, and (a-5) 10,000×; ED-FeCo/TP at magnifications of (b-1) 100×, (b-2) 1000×, (b-3) 3000×, (b-4) 7000×, and (b-5) 10,000×; and ED-FeCo/TP-ArMP15 at magnifications of (c-1) 100×, (c-2) 1000×, (c-3) 3000×, (c-4) 7000×, and (c-5) 10,000×.

Figure 5c presents the surface morphology of the electrocatalyst after Ar MP treatment. Compared with the untreated sample, ED-FeCo/TP-ArMP15 exhibits a more irregular and rough granular texture, with the originally aggregated particles showing partially fractured features. This change likely results from ion bombardment in the highly dissociated microwave plasma, which induces an etching-like effect on the surface. Consequently, the MP-treated surface becomes significantly rougher than that of the untreated sample. Such a roughened surface promotes more specific surface area between the electrolyte and the electrocatalyst, increases the electrochemically active surface area (ECSA), and provides more active sites for the OER, thereby enhancing the catalytic performance. From the SEM analysis, it can be inferred that the MP treatment increases the active surface area of the catalyst, leading to improved OER performance for ED-FeCo/TP-ArMP15 compared with ED-FeCo/TP.

Figure S1 presents the EDS mapping results of (a) TP, (b) ED-FeCo/TP, and (c) ED-FeCo/TP-ArMP15. The pristine TP shows a strong Ti signal, corresponding to the distribution of the Ti metal fibers. After the electrodeposition of FeCo, the ED-FeCo/TP sample exhibits clear Fe and Co signals covering the Ti substrate, while the Ti signal becomes noticeably weaker due to the overlayer of FeCo materials. For ED-FeCo/TP-ArMP15, the Fe and Co distributions appear slightly more dispersed, which can be attributed to surface etching caused by energetic ion bombardment during the MP treatment. Although the Fe and Co signals are slightly weakened, both elements remain uniformly distributed across the Ti fibers.

### 3.3 XPS

In this study, XPS was employed to analyze the effect of MP treatment on the bonding states and oxidation states of the materials. The XPS survey spectra results (Figure 6a–c) indicate that after electrochemical deposition, both ED-FeCo/TP and ED-FeCo/TP-ArMP15 exhibit clear Fe and Co signals, while the Ti signal appears weaker. This reduction in Ti intensity is attributed to the FeCo overlayer covering the underlying Ti metal fibers after deposition. Based on the XPS survey spectra, it can be confirmed that Fe and Co were successfully deposited onto the Ti metal fiber substrate. To examine the variations in the oxidation state and bonding characteristics of Fe, high-resolution XPS analysis of Fe 2p was performed, as illustrated in Figure 6d,e. The Fe 2p XPS spectrum of ED-FeCo/TP (Figure 6d) revealed binding energies of 720.18 eV for Fe 2p_1/2_ and 706.18 eV for Fe 2p_3/2_, with an additional satellite signal observed at 714.58 eV.^17,20,22,55^ As shown in Figure 6d, Fe^2+^ in ED-FeCo/TP was significantly more abundant than Fe^3+^, with Fe^2+^ and Fe^3+^ exhibiting binding energies of 708.60 and 711.38 eV, respectively.^17,20^ After Ar MP treatment (Figure 6e), the Fe^3+^ peak intensity of the ED-FeCo/TP-ArMP15 sample increased slightly, while the Fe^2+^ peak intensity decreases, with binding energies of 709.87 and 712.37 eV for Fe^2+^ and Fe^3+^, respectively.^17,20^ According to Table 1, the Fe^0^ content in ED-FeCo/TP increased significantly from 50.54% to 58.9% after ArMP treatment.^20^ These findings suggest that the Ar MP treatment facilitates the reduction of Fe, thereby increasing the proportion of metallic Fe (Fe^0^). Figure 6f,g shows the high-resolution XPS analysis of the Co 2p orbitals, demonstrating the presence of both Co^2+^ and Co^3+^ oxidation states. The Co 2p spectrum for ED-FeCo/TP (Figure 6f), Co^3+^ is identified by the peaks at 780.87 and 796.39 eV, and Co^2+^ is represented by the peaks at 782.34 and 797.37 eV^21,35^ . As depicted in Figure 6g, the Ar MP-treated ED-FeCo/TP-ArMP15 sample reveals the presence of metallic Co (Co^0^), with signals at 778.33 eV for the Co 2p3/2 orbital and 793.03 eV for the Co 2p_1/2_ orbital.^21,35^ According to Table 2, the metallic Co (Co^0^) proportion increases to 44.11% after Ar MP treatment, demonstrating that Ar MP effectively promotes the reduction of Co to form metallic Co (Co^0^).^35^ From the XPS fine-scan analysis, it can be observed that the sample treated with Ar MP exhibits partial reduction of Fe and Co species, indicating the presence of metallic Fe and Co phases. It is speculated that these metallic phases may enhance the OER catalytic performance by improving the overall electrical conductivity of the electrode, forming metal/metal oxide heterointerfaces that facilitate charge transfer, and serving as precursors that can be transformed into more active oxyhydroxide species during the OER process.^56^

**Figure 6:**
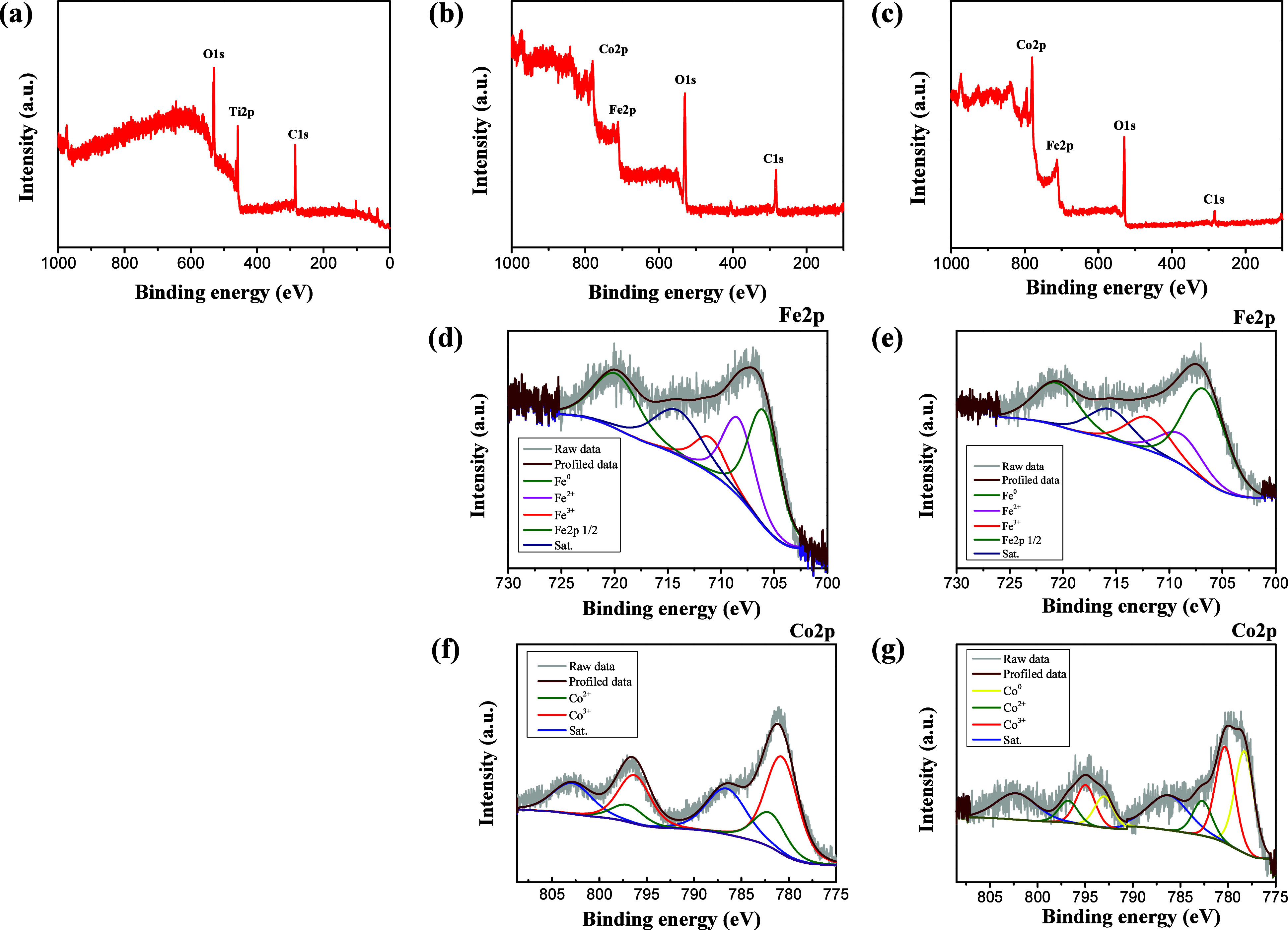
Full survey XPS spectra of (a) TP, (b) ED-FeCo/TP, and (c) ED-FeCo/TP-ArMP15. High-resolution Fe 2p spectra of (d) ED-FeCo/TP and (e) ED-FeCo/TP-ArMP15. High-resolution Co 2p spectra of (f) ED-FeCo/TP and (g) ED-FeCo/TP-ArMP15.

**Table 1:** Proportions of Different Oxidation States of Fe 2p in Each Sample Obtained from XPS Fine Scan Analysis (at. %)

at. %	ED-FeCo/TP	ED-FeCo/TP-ArMP15
Fe^0^	50.54	58.90
Fe^2+^	33.9	19.68
Fe^3+^	15.5	21.40

**Table 2:** Proportions of Different Oxidation States of Co 2p in Each Sample Obtained from XPS Fine Scan Analysis (at. %)

at. %	ED-FeCo/TP	ED-FeCo/TP-ArMP15
Co^0^	N/A	44.11
Co^2+^	10.31	15.03
Co^3+^	89.69	40.85

### 3.4 Electrochemical Measurement

Electrochemical analysis was conducted to evaluate the OER catalytic performance of ED-FeCo/TP and ED-FeCo/TP-ArMP15, and the results are presented in Figure 7 and Table 3. In Figure 7a, the polarization curves obtained from LSV measurements reflect the OER activity. At a current density of 10 mA/cm^2^, the overpotentials of ED-FeCo/TP and ED-FeCo/TP-ArMP15 are 270 mV and 328 mV, respectively. When the current density increases to 100 mA/cm^2^, the overpotentials become 439 mV for ED-FeCo/TP and 459 mV for ED-FeCo/TP-ArMP15. This trend indicates that the overpotential difference between the two samples decreases as the current density increases. At higher voltages, ED-FeCo/TP-ArMP15 can even approach or slightly exceed the current density of ED-FeCo/TP, demonstrating comparable high-current behavior. The Tafel slope analysis shown in Figure 7b reveals values of 276.4 mV/dec for ED-FeCo/TP and 211.1 mV/dec for ED-FeCo/TP-ArMP15, suggesting that the reaction pathway may be altered after plasma treatment. EIS results (Figure 7c) further demonstrate a change in impedance behavior before and after MP treatment. For the untreated ED-FeCo/TP, two semicircles are observed. One corresponds to the film porous structure resistance (*R*_f_ = 0.70 Ω) and the other to the charge-transfer resistance (*R*_ct_ = 1.13 Ω).^20,57^ In contrast, for the ED-FeCo/TP-ArMP15, only a single semicircle associated with *R*_ct_ (1.10 Ω) is present. The disappearance of the film porous structure resistance after plasma treatment may result from localized heating and partial sintering of the FeCo layer onto the Ti fibers during MP exposure, thus reducing the interface resistance. Figure 7d–f) show the CV measurements and the calculation of double-layer capacitance (2*C*_dl_). The 2*C*_dl_ values were obtained by measuring Δ*j* (=*j*_cathodic_ – *j*_anodic_) at different scan rates and plotting them against the scan rate, and the slope of the fitted line corresponds to 2*C*_dl_.^58^ The calculated 2*C*_dl_ values are 2.8 mF/cm^2^ for ED-FeCo/TP-ArMP15 and 0.09 mF/cm^2^ for ED-FeCo/TP, indicating a significant enhancement in capacitive behavior after plasma treatment. The ECSA was further estimated using the following equation^43,59^

4 $$ECSA=\frac{C_{dl}}{C_{s}}$$

*C*_s_ represents the specific capacitance of a smooth surface (typically 0.04 mF/cm^2^ for alkaline solution). After calculation, the ECSA values were 35.0 cm^2^ for ED-FeCo/TP-ArMP15 and 11.3 cm^2^ for ED-FeCo/TP. This notable increase suggests that MP treatment generates more active sites, consistent with the SEM results, where plasma-induced ion bombardment produces surface cracking and roughening. The enhanced roughness increases the specific surface area, providing a greater number of accessible active sites and consequently improving OER catalytic activity.

**Figure 7:**
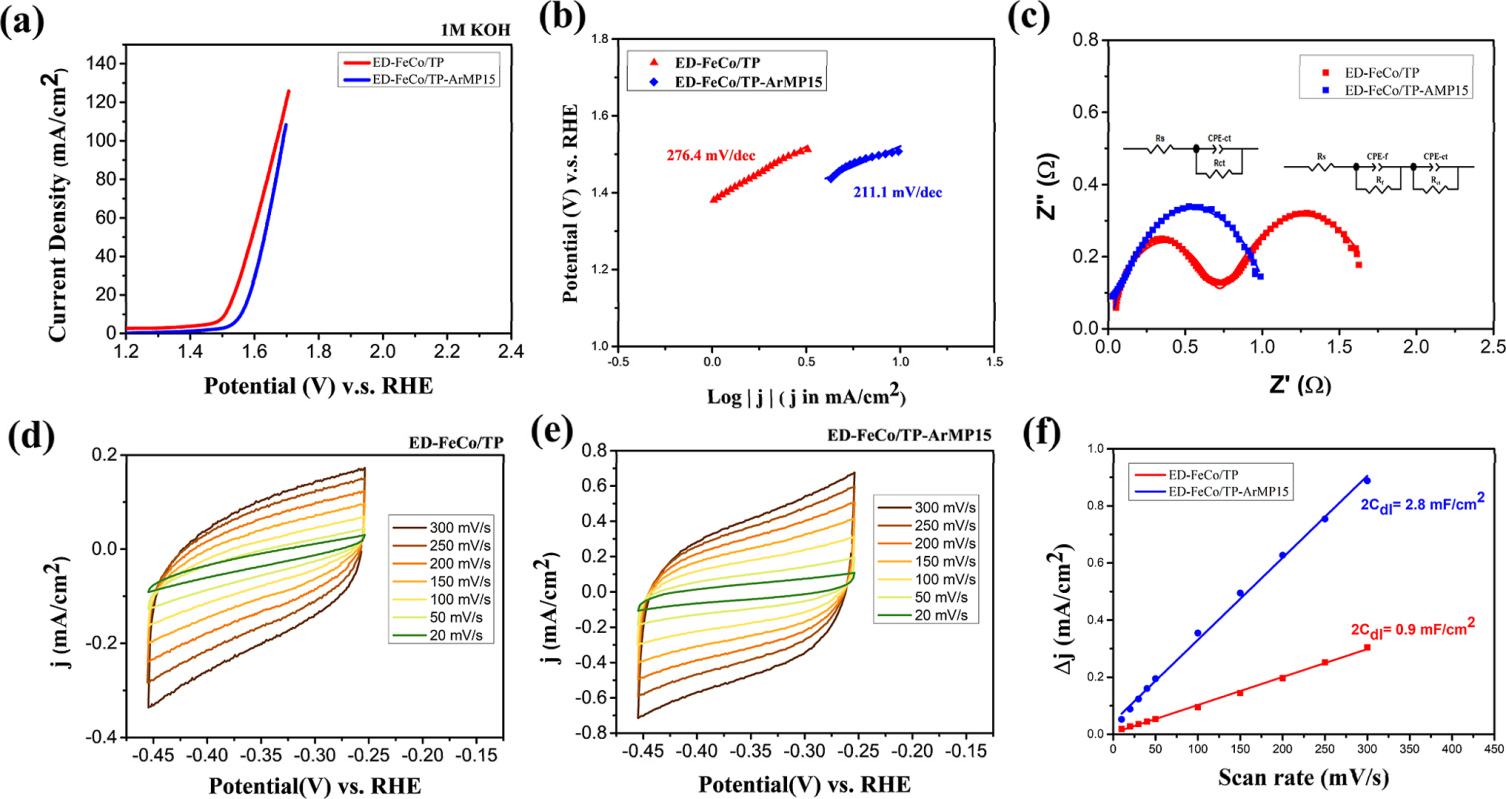
(a) LSV polarization curves for the OER in 1 M KOH. (b) Tafel slope diagrams. (c) Nyquist plots. CV measurement of (d) ED-FeCo/TP and (e) ED-FeCo/TP-ArMP15 at various scan rates and (f) electrochemical double-layer capacitance.

**Table 3:** Electrochemical Analysis and Calculated Results of ED-FeCo/TP and ED-FeCo/TP-ArMP15

electrocatalyst	overpotential (mV) @10 mA/cm^2^	overpotential (mV) @100 mA/cm^2^	Tafel slope (mV/dec)	*R*_f_ (Ω)	*R*_ct_ (Ω)	2*C*_dl_ (mF/cm^2^)	ECSA (cm^2^)
ED-FeCo/TP	270	439	276.4	0.70	1.13	0.90	11.3
ED-FeCo/TP-ArMP15	328	459	211.1	N/A	1.10	2.80	35.0

### 3.5 Performance of AEMWE

To investigate the practical application of the ED-FeCo/TP-ArMP15 anode electrocatalyst in anAEMWE, a full-cell study was conducted. The AEM electrolyzer employed in this work is a symmetric cell, consisting of an AEM at the center, sandwiched by the catalyst-coated PTLs, bipolar flow plates, and heating plates used for temperature control. For the cathodic PTL, Ru/CP was used, while ED-FeCo/TP and ED-FeCo/TP-ArMP15 were adopted as the anodic PTLs to assemble the AEM full cells. The influence of MP treatment on the anode PTL was then investigated. During AEMWE operation, a cathode-dry mode was adopted, meaning that the electrolyte was only supplied to the anode side. In this study, 1 M KOH was used as the electrolyte, which diffused through the membrane to reach the cathode, where HER occurred. The cathode-dry operation theoretically produces hydrogen with lower humidity and lower alkalinity, which facilitates subsequent hydrogen processing or utilization while reducing system cost and complexity.^41–43^

Figure 8 shows the polarization curves of the Ru/CP(−)//ED-FeCo/TP­(+) and Ru/CP(−)//ED-FeCo/TP-ArMP15­(+) systems, along with their performance variations at different operating temperatures. From the polarization curves, it can be observed that increasing the temperature enhances the overall performance of the AEMWE system. This improvement is attributed not only to the increased reaction kinetics of water electrolysis but also to the alleviation of mass transport limitations such as electrolyte diffusion and wetting on the cathode side in the cathode-dry operation.^17,43^ Notably, at low current densities, the untreated system, Ru/CP(−)//ED-FeCo/TP­(+), exhibits slightly higher or comparable performance to the Ru/CP(−)//ED-FeCo/TP-ArMP15­(+) system. However, as the current density increases, the Ru/CP(−)//ED-FeCo/TP-ArMP15­(+) cell requires a lower operating voltage to achieve the same current density, indicating that the MP-treated system delivers better performance under high-current conditions. This trend is consistent with the LSV polarization results, where MP-treated samples demonstrate superior catalytic activity at higher current densities.

**Figure 8:**
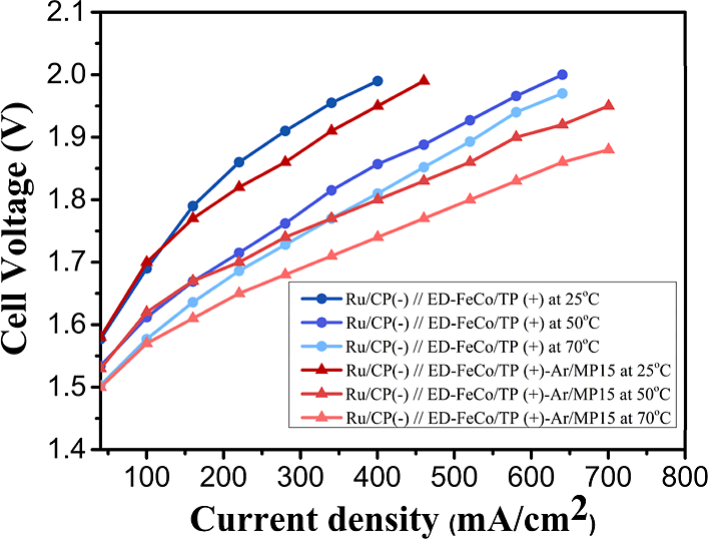
Polarization curve of AEMWE at 25 °C, 50 and 70 °C for Ru/CP(−)//ED-FeCo/TP­(+) and Ru/CP(−)//ED-FeCo/TP-ArMP15­(+).

In addition to the polarization curves, the key characteristics of the cathode-dry AEMWE system were further analyzed, including the specific energy consumption (by volume and by weight) and the energy efficiency (η), as summarized in Table 4. The power supply voltage (*V*_ps_) refers to the total voltage applied to the electrolyzer and the external circuit, while the cell voltage (*V*_cell_) represents the actual voltage across the AEMWE cell, which was used to plot the polarization curves. The specific energy consumption (by volume) was calculated according to eq 5 ^21,60,61^

5 $$specific\;energy\;consumption=\frac{I\times V_{ps}}{P_{H_{2}}}$$

where *I* is the total current (A) and $P_{H_{2}}$ is the measured hydrogen production rate (mL/min). The specific energy consumption (by weight) was further calculated using the conversion factor that 1 kg of H_2_ gas occupies 11.2 m^3^ at normal temperature and pressure (*NTP*). The energy efficiency (η) was determined based on the thermo-neutral voltage (*V*_TN_), calculated as follows^60,61^

6 $$V_{TN}=\frac{\Delta H}{n\cdot F}$$

where Δ*H* is the enthalpy of hydrogen (286 kJ/mol), n is the number of electrons involved in the reaction (*n* = 2 for H_2_ production), and F is the Faraday constant (96,500 C/mol).

**Table 4:** Performance Parameters of AEMWE Cell Test with Each Electrocatalyst

electrocatalysts	temperature (°C)	current density (mA/cm^2^)	power supply voltage (V)	cell voltage (V)	H_2_ production rate (mL/min)	specific energy consumption (volume) (kWh/m^3^)	specific energy consumption (weight) (kWh/kg)	energy efficiency (from *V*_TN_) (%)
Ru/CP(−)//ED-FeCo/TP (+)	25	300	1.97	1.93	12	4.10	45.97	75.13
	50	500	1.97	1.91	19	4.32	48.39	75.13
	70	500	1.94	1.88	18	4.49	50.30	76.29
Ru/CP(−)//ED-FeCo/TP-ArMP15(+)	25	300	1.89	1.88	11.5	4.11	46.02	78.31
	50	500	1.87	1.86	17	4.58	51.33	79.14
	70	500	1.8	1.79	18	4.17	46.70	82.22

The overall energy efficiency was then calculated using eq 7 ^43,60,61^

7 $$\eta =\frac{V_{TN}}{V_{ps}}$$

From these calculations, both the Ru/CP(−)//ED-FeCo/TP­(+) and Ru/CP(−)//ED-FeCo/TP-ArMP15­(+) systems exhibit improved performance at elevated temperatures, particularly at 70 °C. At 500 mA/cm^2^ and 70 °C, the MP-treated system, Ru/CP(−)//ED-FeCo/TP-ArMP15­(+), required a lower applied voltage (*V*_ps_ = 1.80 V), leading to reduced specific energy consumption (4.17 kWh/m^3^ and 46.70 kWh/kg) compared with the untreated system (4.49 kWh/m^3^ and 50.30 kWh/kg). Furthermore, the energy efficiency of the Ru/CP(−)//ED-FeCo/TP-ArMP15­(+) system reached 82.22%, higher than that of the untreated cell (76.29%). The stability results, shown in Figure S3, demonstrate that the Ru/CP(−)//ED-FeCo/TP-ArMP15­(+) system maintained stable operation at 25 °C and 200 mA/cm^2^, with only minor voltage variation in the initial stage and a steady profile after 5 h. The anodic PTL, ED-FeCo/TP-ArMP15­(+), also exhibited excellent stability in a three-electrode configuration at 25 °C and 10 mA/cm^2^, maintaining a constant potential around 0.55 V (Figure S4).

Overall, both the polarization and energy analyses confirm that the MP-treated system exhibits superior hydrogen generation efficiency, lower specific energy consumption, higher energy efficiency, and stable long-term performance, demonstrating the beneficial role of microwave plasma treatment on the OER PTL and its practical potential in cathode-dry AEMWE systems.

## 4 Conclusion

The ED-FeCo/TP-ArMP15 catalyst was successfully developed through the electrochemical deposition of FeCo materials on TP, followed by ArMP treatment, resulting in significantly enhanced OER performance and durability. Electrochemical analysis revealed that ED-FeCo/TP-ArMP15 possessed superior catalytic properties, including an overpotential of 459 mV@100 mA/cm^2^, a reduced Tafel slope (211.1 mV/dec), a higher double-layer capacitance (2.80 mF/cm^2^), and a lower charge transfer resistance (1.10 Ω). These attributes to enhanced reaction kinetics with an increased ECSA and reduced resistance. Moreover, the reduction effect introduced by ArMP treatment resulted in the generation of a substantial amount of metallic Fe^0^ and Co^0^ species in ED-FeCo/TP-ArMP15. When used as the anode PTL in a cathode-dry AEMWE system operating at different temperatures, the efficiency at a current density of 500 mA/cm^2^ increased in all cases. The system with MP-treatment (Ru/CP(−)//ED-FeCo/TP-ArMP15­(+)) exhibited superior performance at higher current densities compared to the untreated system (Ru/CP(−)//ED-FeCo/TP­(+)), along with lower specific energy consumption and higher energy efficiency. This improvement can be ascribed to the morphology changes induced by ArMP treatment, which generated surface defects and active sites, ultimately enhancing the OER efficiency. The effectiveness of ArMP treatment in optimizing electrocatalytic performance has been demonstrated through electrochemical tests and practical applications in electrolysis cells, highlighting its potential as a promising strategy to enhance hydrogen production technologies.

## Supplementary Material



## Notes

Supporting Information includes EDS mapping results, XRD results, durability test of AEM and ED-FeCo/TP-ArMP15 in a three-electrode system, and the comparison of several FeCo-based OER electrocatalysts (PDF)
