# Supplementary material for: Argon Microwave Plasma Processed Electrodeposited FeCo Catalysts on Ti Paper as an Anode Porous Transport Layer (PTL) for Cathode-Dry Anion Exchange Membrane Water Electrolysis (AEMWE)
Source: ACS Omega. 2026 Mar 11;11(11):17292–301. doi: 10.1021/acsomega.5c09854 (PMC13019374; doi:10.1021/acsomega.5c09854)
Supplement: Supplementary file 1 [file ao5c09854_si_001.pdf]

Supporting information

Argon Microwave Plasma Processed Electrodeposited FeCo Catalysts on Ti Paper as an Anode Porous Transport Layer (PTL) for Cathode-Dry Anion Exchange Membrane Water Electrolysis (AEMWE)

Hsing-Chen Wu[1,#], Shuo-En Yu[2,#], I-Chun Cheng [3], Jian-Zhang Chen[1,2,4,5*]

[1] *Institute of Applied Mechanics, National Taiwan University, Taipei City 106319, Taiwan*

[2] *Graduate School of Advanced Technology, National Taiwan University, Taipei City 106319, Taiwan*

[3] *Graduate Institute of Photonics and Optoelectronics and Department of Electrical Engineering, National Taiwan University, Taipei City 106319, Taiwan*

[4]*Advanced Research Center for Green Materials Science and Technology, National Taiwan University, Taipei City 106319, Taiwan*

[5]*Research Center for Applied Sciences, Academia Sinica, Taipei City 115201, Taiwan*

*Corresponding Authors:

jchen@ntu.edu.tw (J.Z.C.); Tel.: +886-2-3366-5694 (J.Z.C.)

# The authors contribute equally to this work.

Figure S1. EDS mapping results of (a) TP, (b) ED-FeCo/TP, (c) ED-FeCo/TP-ArMP15.
Figure S2. XRD of TP, ED-FeCo/TP, and ED-FeCo/TP-ArMP15.
Figure S3. Durability test of catalyst in AEM water electrolyzer at 25 °C.
Figure S4. Durability test of catalyst in electrochemical long-term measurement.
Table S1. Comparison of several FeCo-based OER electrocatalyst electrodes with this work.

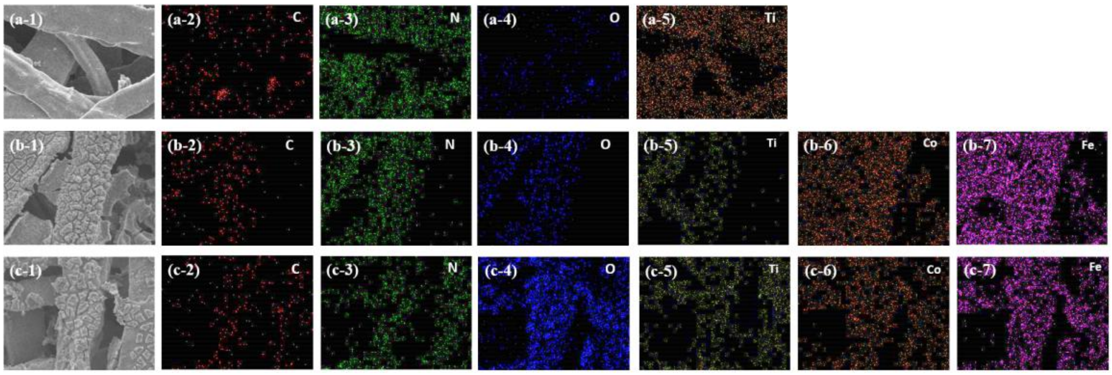

Figure S1. EDS mapping results of (a) TP, (b) ED-FeCo/TP, and (c) ED-FeCo/TP-ArMP15. (1) SEM image; and (2)-(7) correspond to the elemental distributions of C, N, O, Ti, Co, and Fe, respectively.

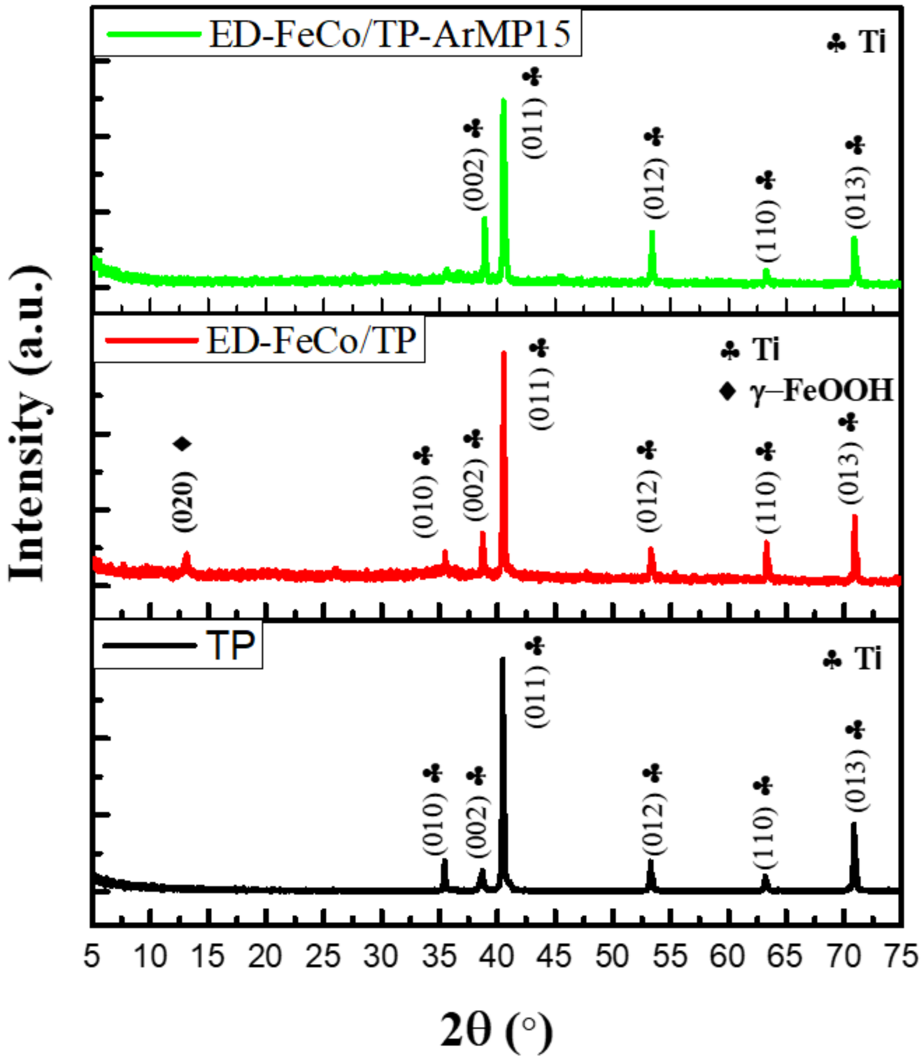

Figure S2. XRD of TP, ED-FeCo/TP, and ED-FeCo/TP-ArMP15.

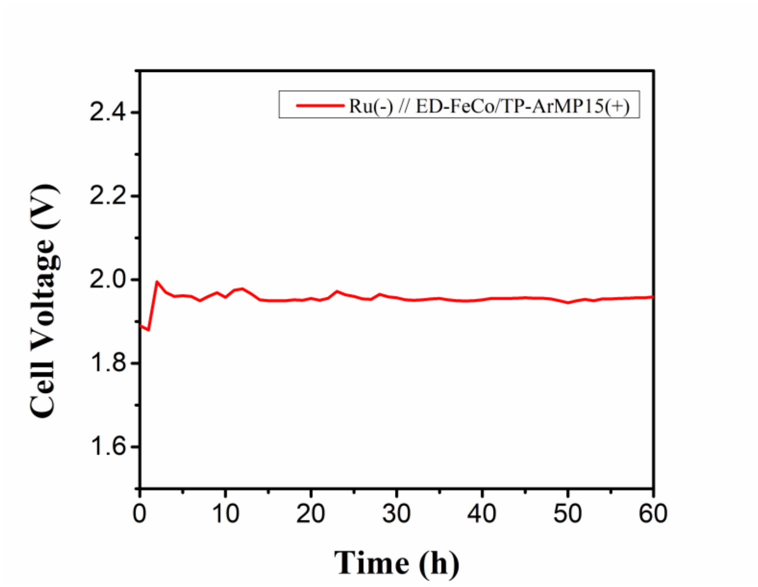

Figure S3. Durability test of catalyst in AEMWE at 25 °C

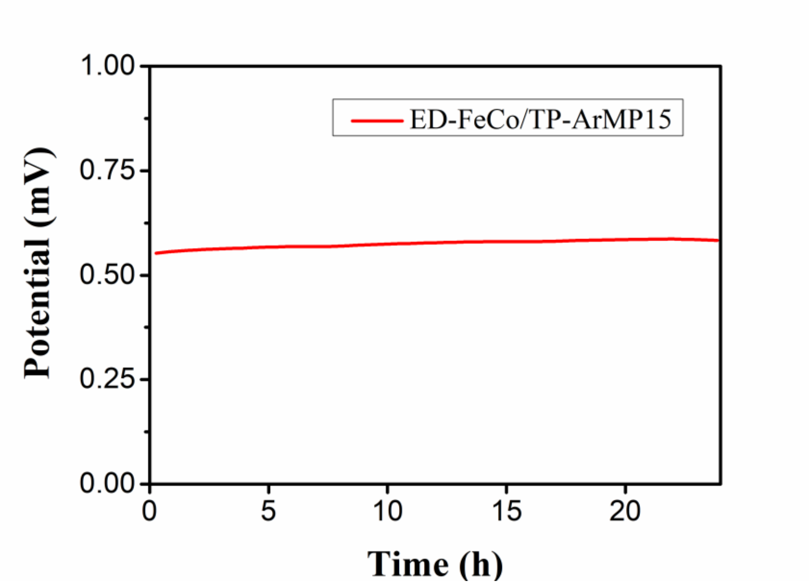

Figure S4. Durability test of catalyst in electrochemical measurement

Table S1. Comparison of several FeCo-based OER electrocatalyst electrodes with this work.

Electroatalyst	Electrolyte	Overpotential (mV@ mA/cm^2)	Reference
ED-FeCo/TP<br>ED-FeCo/TP-ArMP15	1 M KOH	270@10<br>328@10	This work
Co/Fe 64<br>Co/Fe 32	0.1 M KOH	496@10<br>486@10	1
$CoFe_2O_4$ powders (agar-agar)<br>$CoFe_2O_4$ powders (Gelatin)	1 M KOH	360@10<br>435@10	2
$Co_{0.20}Fe_{0.80}OOH$	1 M KOH	383@10	3
FeOOH/Co/FeOOH HNTAs-NF	1 M NaOH	~250@20	4
Fe-CoOOH/G	1 M KOH	330@10	5
CoFe-LDH<br>CoFe-LDH@g-C_3N_4	1 M KOH	314@10<br>275@10	6
CoFe-LDH	1 M KOH	400@10	7
CoFe-MWCNTs	1 M KOH	300@10	8
FeCoO35	1 M KOH	331@10	9
Fe–Co–P alloy	1 M KOH	252@10	10

Reference

(1) Grewe, T.; Deng, X.; Tüysüz, H. Influence of Fe Doping on Structure and Water Oxidation Activity of Nanocast Co3O4. *Chemistry of Materials* **2014**, *26* (10), 3162-3168. DOI: 10.1021/cm5005888.

(2) Ferreira, L. S.; Silva, T. R.; Santos, J. R. D.; Silva, V. D.; Raimundo, R. A.; Morales, M. A.; Macedo, D. A. Structure, magnetic behavior and OER activity of CoFe2O4 powders

obtained using agar-agar from red seaweed (Rhodophyta). *Materials Chemistry and Physics* **2019**, *237*. DOI: 10.1016/j.matchemphys.2019.121847.
(3) Inohara, D.; Maruyama, H.; Kakihara, Y.; Kurokawa, H.; Nakayama, M. Cobalt-Doped Goethite-Type Iron Oxyhydroxide (alpha-FeOOH) for Highly Efficient Oxygen Evolution Catalysis. *ACS Omega* **2018**, *3* (7), 7840-7845. DOI: 10.1021/acsomega.8b01206 From NLM PubMed-not-MEDLINE.
(4) Feng, J. X.; Xu, H.; Dong, Y. T.; Ye, S. H.; Tong, Y. X.; Li, G. R. FeOOH/Co/FeOOH Hybrid Nanotube Arrays as High-Performance Electrocatalysts for the Oxygen Evolution Reaction. *Angew Chem Int Ed Engl* **2016**, *55* (11), 3694-3698. DOI: 10.1002/anie.201511447 From NLM PubMed-not-MEDLINE.
(5) Han, X.; Yu, C.; Zhou, S.; Zhao, C.; Huang, H.; Yang, J.; Liu, Z.; Zhao, J.; Qiu, J. Ultrasensitive Iron-Triggered Nanosized Fe–CoOOH Integrated with Graphene for Highly Efficient Oxygen Evolution. *Advanced Energy Materials* **2017**, *7* (14). DOI: 10.1002/aenm.201602148.
(6) Arif, M.; Yasin, G.; Shakeel, M.; Mushtaq, M. A.; Ye, W.; Fang, X.; Ji, S.; Yan, D. Hierarchical CoFe-layered double hydroxide and g-C3N4 heterostructures with enhanced bifunctional photo/electrocatalytic activity towards overall water splitting. *Materials Chemistry Frontiers* **2019**, *3* (3), 520-531. DOI: 10.1039/c8qm00677f.
(7) Feng, L.; Li, A.; Li, Y.; Liu, J.; Wang, L.; Huang, L.; Wang, Y.; Ge, X. A Highly Active CoFe Layered Double Hydroxide for Water Splitting. *Chempluschem* **2017**, *82* (3), 483-488. DOI: 10.1002/cplu.201700005 From NLM PubMed-not-MEDLINE.
(8) Ali, Z.; Mehmood, M.; Ahmed, J.; Majeed, A.; Thebo, K. H. CVD grown defect rich-MWCNTs with anchored CoFe alloy nanoparticles for OER activity. *Materials Letters* **2020**, *259*. DOI: 10.1016/j.matlet.2019.126831.
(9) Mohanty, R. I.; Mukherjee, A.; Basu, S.; Bhanja, P.; Jena, B. K. Iron Cobalt Phosphonate Derived Heteroatom Doped Metal Oxides as Superior Electrocatalysts for Water Oxidation Reaction. *ChemCatChem* **2023**, *15* (20). DOI: 10.1002/cctc.202300731.
(10) Liu, K.; Zhang, C.; Sun, Y.; Zhang, G.; Shen, X.; Zou, F.; Zhang, H.; Wu, Z.; Wegener, E. C.; Taubert, C. J.; et al. High-Performance Transition Metal Phosphide Alloy Catalyst for Oxygen Evolution Reaction. *ACS Nano* **2018**, *12* (1), 158-167. DOI: 10.1021/acsnano.7b04646 From NLM PubMed-not-MEDLINE.
